# Thalamo-mesencephalic Branches of the Posterior Cerebral Artery: a 3D Rotational Angiography Study

**DOI:** 10.1007/s00062-024-01418-y

**Published:** 2024-04-26

**Authors:** Maximilian Rauch, Joachim Berkefeld, Madleen Klonowski, Elke Hattingen, Stefan Weidauer

**Affiliations:** University Hospital, Institute for Neuroradiology, Goethe University Frankfurt, Theodor Stern Kai 7, 60590 Frankfurt am Main, Germany

**Keywords:** Neuroanatomy, Digital subtraction angiography, Posterior cerebral artery, Thalamus, Mesencephalon

## Abstract

**Purpose:**

The thalamo-mesencephalic (TM) branches of the posterior cerebral artery (PCA) supply critical structures. Previous descriptions of these vessels are inconsistent and almost exclusively rely on cadaver studies. We aimed to provide a neuroradiological description of TM vessels in vivo based on routine 3D rotational angiographies (3D-RA).

**Methods:**

We analyzed 3D-RAs of 58 patients with pathologies remote from the PCA. PCA-origins were considered. Delineation, origin and number of branches of the collicular artery (CA), the accessory CA (ACA), the posterior thalamoperforating artery (PTA), the thalamogeniculate artery (TGA), and the posterior medial (PMCA) and lateral (PCLA) choroid arteries were assessed. The PTAs were categorized based on Percheron’s suggested classification.

**Results:**

A CA was identified in 84%, an ACA in 20%. The PTA was delineated in 100%. In 27%, PTA anatomy had features of several Percheron types (*n* = 7) or vessels emanating from a net like origin (*n* = 9). 26% had a type IIb PTA. A fetal type PCA origin with hypoplastic ipsilateral P1 was observed in 5 cases with type IIa (*n* = 2) or type IIb (*n* = 3) PTAs originating from contralateral P1. The TGA was identified in 85% of patients, with ≥ 2 branches in 67%. The PMCA was delineable in 41%, the PLCA in 100%.

**Conclusion:**

The prevalence of a proper “Artery of Percheron” type IIb PTA seems to be higher than previously reported. A fetal type P1-origin may be predictive of a type IIa/b PTA emanating from contralateral P1. 3D-RA may be useful for planning PCA interventions, as impairment of TM branches is a severe risk.

## Introduction

The thalamus and mesencephalon receive blood supply from the thalamo-mesencephalic (TM) branches of the posterior cerebral artery (PCA). While the thalamus is embedded in functional brain connectivity, mediating motor, sensory, coordinative, memory, cognitive and behavioral functions [1–3], the mesencephalon modulates extrapyramidal motor function, auditory processing and eye movement control [4]. Moreover, the mesencephalon contains both ascending and descending sensory and motor fiber tracts and is involved in the mechanisms of consciousness and sleep [4].

The anatomy of TM vessels has been barely characterized by in vivo methods, i.e. digital subtraction angiography, computed tomography angiography or 1.5 T and 3 T magnetic resonance angiography, as these methods were considered to have too low resolution to image small caliber branches [5].

As a result, most research on the anatomy of TM vessels has relied on cadaver brain examinations, resulting in inconsistent findings [6]. However, understanding the anatomy of TM vessels is crucial, as damage to these branches during trauma or neurosurgical and neurointerventional procedures can have severe repercussions for the patient. The aim of our study is to provide an in vivo neuroradiological illustration of TM vessels using routine 3D rotational digital subtraction angiography (3D-RA).

## Material and Methods

**Fig. 1:**
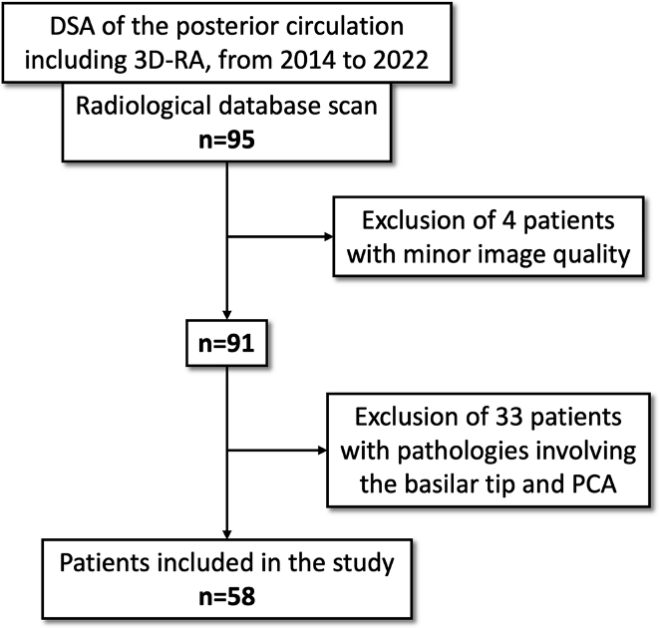
Study flowchart describing the data procurement and patient selection process intended for retrospective analysis

This study was a single-center, retrospective analysis that was approved by the local ethics committee of the faculty of medicine. We conducted a review of our institutional radiological database to identify patients who underwent diagnostic digital subtraction angiography (DSA) of the posterior circulation from 2014 to 2022, which included routine 3D-RA runs. We collected data from a total of 95 patients, after excluding 4 datasets with minor image quality issues resulting from incomplete contrast filling or movement artifacts. We further excluded 33 datasets from patients with pathologies affecting the basilar tip and PCA. Datasets of 58 patients with adequate image quality and pathologies remote from the PCA were selected for further evaluation (Fig. 1).

DSA examinations were performed on an Axiom Artis zee biplane neuroradiologic angiography system (Siemens Healthineers, Erlangen, Germany). The angiographic system comes equipped with a flat-panel detector featuring 2480 × 1920 pixels (2k matrix) and a pixel pitch of 154 µm × 154 µm. Indications for DSA and 3D-RA were justified by suspected pathologies like aneurysms, arteriovenous malformations or stenosis. The 3D-RA comprised of a native rotational scan followed by a contrast-enhanced scan after injecting 20 ml of nonionic contrast material (Ultravist 240, Bayer, Berlin, Germany) into the dominant vertebral artery using a 3 ml/s flow rate. An automated injector (Accutron HP‑D, Medtron, Saarbrücken, Germany) was used for all examinations. The injection was initiated 2s before the start of the contrast-enhanced run to obtain 133 images in each run with a scan time of 5s. The flat panel CT scanner achieves 3.0 lp/mm without binning and 1.5 lp/mm with 2 × 2 binning in high-resolution modes. The reconstructed 3D-RA data utilized standard volume rendering and flat panel CT algorithms. Multiplanar reconstructions (MPR) with a section thickness of 0.5 mm, maximum intensity projections (MIP) with section thicknesses ranging from 2 to 20 mm, and volume rendering reconstructions with colored window and threshold settings were utilized to scrutinize the TM vessels.

**Fig. 2:**
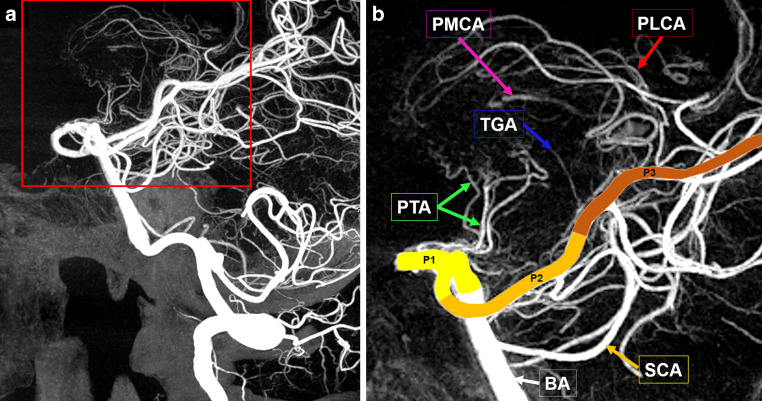
**a** Sagittal 3D rotational angiography maximum intensity projection (MIP) of the intracranial posterior arterial circulation, red square delineating magnification in Fig. 2b. **b** sagittal MIP of the proximal posterior cerebral artery (PCA) and its branches. *BA* basilar artery; *PLCA* posterior lateral choroidal artery; *PMCA* posterior medial choroidal artery; *PTA* posterior thalamoperforating arteries; *SCA* superior cerebellar artery; *TGA* thalamogeniculate artery; *P1–P3* segments of the PCA

**Fig. 3:**
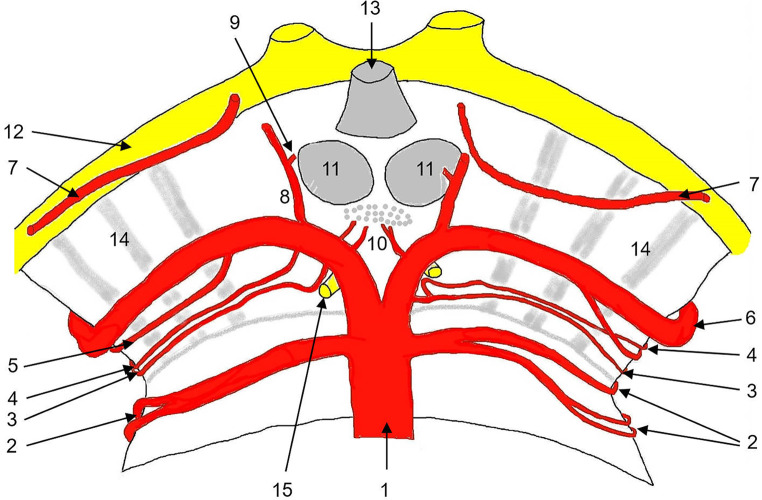
Superficial course of the arteries of the upper pons and mesencephalon. 1: basilar artery; 2: superior cerebellar artery, lateral branches arises inferior, medial branches arises superior; 3: collicular artery; 4: accessory collicular artery; 5: posterior medial choroidal artery; 6: posterior cerebral artery; 7: anterior choroidal artery; 8: posterior communicating artery; 9: anterior thalamoperforating artery (tuberothalamic artery); 10: posterior thalamoperforating arteries; 11: mammillary bodies; 12: optic tract; 13: hypophyseal stalk; 14: cerebral peduncles; 15: oculomotor nerve

**Fig. 4:**
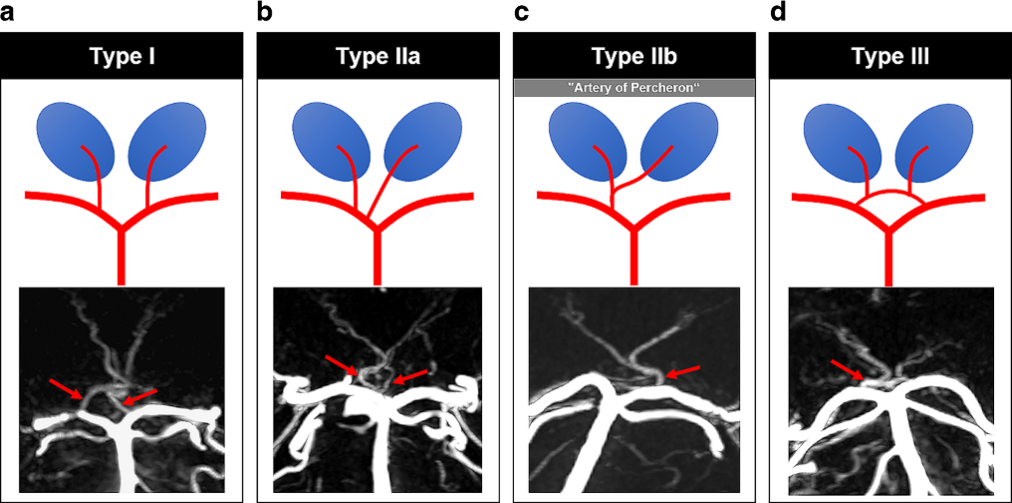
Percheron’s classification of the posterior thalamoperforating arteries (PTA). **a** type 1, bilateral branches (arrows) originating from the P1-segment of the posterior cerebral artery (PCA). **b** type 2a, unilateral branches (arrows). **c** type 2b, unilateral common trunk of the PTA, i.e. proper “artery of Percheron” (arrow). **d** bilateral branches with common anastomosing origin (arrow)

Volume rendered and CT-like MPR and MIP images were then investigated in consensus by two experienced reviewers using a workstation with a Centricity© RIS‑I 7 viewer (GE Healthcare, Chicago, USA). PCA-origins were considered. Delineation, origin and number of branches of the collicular artery (CA), the accessory CA (ACA), the posterior thalamoperforating artery (PTA), the thalamogeniculate artery (TGA), and the posterior medial (PMCA) and lateral (PCLA) choroidal arteries were assessed (Figs. 2 and 3). Synonyms of these vessels are listed in Table 1. The PTAs were categorized based on Percheron’s suggested classification (Fig. 4).

**Table 1:** Thalamo-mesencephalic vessels and synonyms

Vessel	Synonyms	
Collicular artery	Artery of the lamina tecti	*Short and long circumflex arteries*^a^
	Quadrigeminal artery	
	Pedunculoquadrigeminal artery	
Accessory collicular artery	–	
Tuberothalamic artery	Anterior thalamoperforating artery	
	Polar artery	
	Iinferior thalamic peduncle	
	Anterior thalamosubthalamic paramedian artery	
	Premamillary branch of thalamotuberian pedicle	
Posterior thalamoperforating arteries	Paramedian arteries	
	Posterior thalamoperforating arteries	
	Thalamoperforating pedicle	
	Deep interpeduncular artery	
	Retromamillary pedicle	
	Posterior thalamosubthalamic artery	
Thalamogeniculate artery	Thalamogeniculate pedicle	
	Inferolateral branches	
Posterior medial and lateral choroidal arteries	Medial and lateral branches of the posterior choroidal artery	

^a^If several branches supplying the brain stem arise from the P1- and P2-segments, these vessels are also referred to as short and long circumflex arteries

## Results

Patient demographics and the pathologies requiring angiography are shown in Table 2. The angiography was most commonly performed due to ruptured or incidental aneurysms.

**Table 2:** Patients’ demographics

Age (years)	Mean 55, range 16–85
Male (*n*)	21
Female (*n*)	37
(suspected) Aneuryms (*n*)	45
AVM (*n*)	12
Stenosis (*n*)	1

**Fig. 5:**
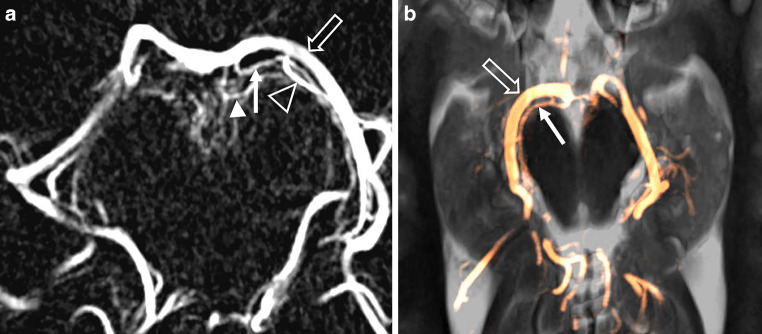
**a** 3D rotational digital subtraction angiography, axial maximum intensity projection (MIP) of the posterior cerebral artery (PCA, open arrow) with collicular artery (CA, arrow), accessory CA (arrowhead) and posterior medial choroidal artery (PMCA, open arrowhead). **b** axial MIP of the PCA (open arrow) and the CA (arrow) projected onto axial T2 weighted MR images

The CA artery was identified in 84% of cases, with axial MIP reconstructions being the optimal means of identifying its course around the midbrain. In most patients, the course of the vessel reaching the quadrigeminal plate was identified (Fig. 5). The ACA, which typically branches from the proximal segment of the CA, was identified in only 20% of the patients in whom the AC was delineated.

**Fig. 6:**
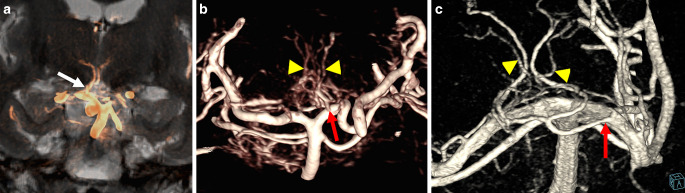
Artery of Percheron. **a** coronal maximum intensity projection (MIP) of the artery of Percheron (arrow) projected onto coronal T2 weighted MR image. **b** posterior thalamoperforating arteries (PTA, arrowheads) emanating from a netlike unilateral structure (arrow). **c** volume rendering of the basilar tip and the P1-segments showing the artery of Percheron (arrow) and accessory small penetrating branches (arrowheads) originating from a netlike structure surrounding the basilar tip and the right P1-segment

The PTA was delineated in 100%, classification according to Percheron is shown in Table 2. 26% had a type IIb PTA, commonly known as the “Artery of Percheron” (Fig. 6a). In 27%, PTA anatomy had features of several types (*n* = 7) or vessels emanating from a network-like origin (*n* = 9; Fig. 6b, c). Coronal MIP reconstructions proved to be the most effective for identifying the PTAs.

A fetal type PCA origin with small caliber hypoplastic ipsilateral P1 was observed in 5 cases. These patients presented with a type IIa (*n* = 2) or type IIb (*n* = 3) PTAs that originated from the contralateral P1 segment.

The TGA was identified in 85% of patients, in 67% of cases, ≥ 2 branches were delineable.

Additionally, 5 cases had a fetal-type PCA origin with a small-caliber hypoplastic ipsilateral P1. These patients presented with either type IIa (*n* = 2) or type IIb (*n* = 3) PTA that originated from the contralateral P1 segment.

In 85% of patients, the TGA was identified, and in 67% of cases, ≥ 2 branches could be delineated.

The PMCA was delineable in 41% of patients, whereas the PLCA was easily identified in 100% due to its course to the choroid plexus of the lateral ventricle. Table 3 provides a summary of the delineation of TM vessels.

**Table 3:** 3D rotational angiography findings

Vessel	Delineation (%)	≥ 2 branches (%)
**Collicular artery (CA)**	49/58 (84)	–
**Accessory collicular artery (ACA)**	10/49 (20)	
**Posterior thalamoperforating artery (PTA)**	58/58 (100)	–
*Type*		
I	12/58 (21)	
IIa	5/58 (9)	
IIb	15/58 (26)	
III	10/58 (17)	
**No classification**	16/58 (27)	
**Thalamogeniculate artery (TGA)**	49/58 (85)	33/49 (67)
**Posterior medial choroidal artery (PMCA)**	24/58 (41)	11/24 (46)
**Posterior lateral choroidal artery (PLCA)**	58/58 (100)	29/58 (50)

## Discussion

**Fig. 7:**
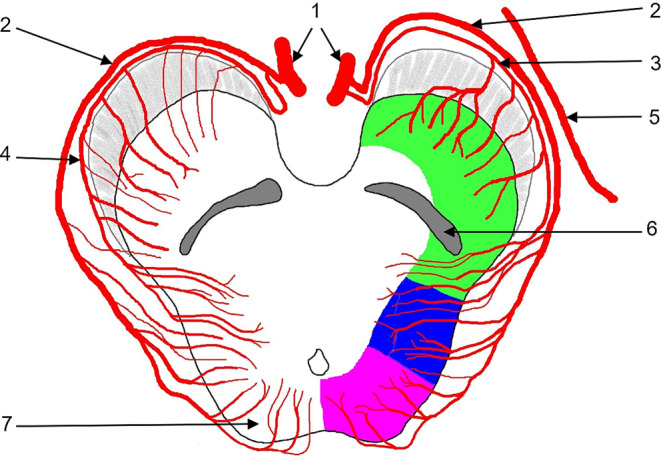
Transverse section of the upper mesencephalon and course of the collicular artery (CA) and lateral vascular territories of the midbrain. 1: posterior cerebral artery (PCA, P1-segment); 2: CA; 3, 4: accessory CA (ACA), left ACA originating from the P1-segment, right ACA originating from the CA; 5: anterior choroidal artery (ACHA); 6: substantia nigra; 7: tectal plate; green: anterolateral group (CA, ACHA, posterior medial choroidal artery [PMCA]); blue: lateral group (CA, PMCA, PCA); purple: posterior group (CA, PMCA)

The most proximal TM branch of the PCA is the CA. The CA has rather been underappreciated in the literature. Some authors refer this vessel to as the long circumflex artery (Table 1; [6]). The CA either arises from either P1 or the proximal P2 PCA-segment close to the origin of the posterior communicating artery, encircles the midbrain and extends to the quadrigeminal plate (Figs. 5 and 7; [7]). It supplies the superior and inferior colliculi, structures critical for central auditory and visual processing. Additionally, its territory includes the cerebral peduncle, the tegmentum, the medial geniculate body, and the pulvinar [6]. The terminal CA anastomoses with branches of the superior cerebellar artery forming an extensive arterial network on the superior colliculi [8, 9]. The diameter of the CA is reported to be 0.5–0.8 mm [6, 10].

The ACA, arises from the proximal CA or originates separately from the P1 segment [6]. It accompanies the CA in its course, inferior to the PCA and reaches the medial geniculate body. The substantia nigra may also receive blood supply from the ACA [6]. The number of ACA branches per hemisphere has been observed to vary from one to three [11]. In a study conducted by Zeal and Rhoton [8], it was found that 66% of post-mortem examined hemispheres demonstrated the presence of an ACA, which commonly terminated at the posterolateral edge of the cerebral peduncle. Another cadaver study identified the ACA in 43% [12].

If several branches supplying the brain stem arise from the P1- and P2-segments, these vessels were also referred to as short and long circumflex arteries by some authors. The short circumflex arteries course to the interpeduncular fossa and posterior perforating substance, whereas the long circumflex arteries encircle the brainstem to reach the quadrigeminal and colliculi.

To the best of our knowledge, there are no documented cases in the literature of isolated impairment of either of these vessels, such as occlusion. However, involvement of the CA or ACA in posterior circulation strokes is not uncommon and can cause harm to critical dependent mesencephalic structures, leading to a poor prognosis (Fig. 7; [13]).

We identified the CA in the majority of patients. In only 20% of patient in which an CA was identified, an ACA was delineable. However, the reported diameter of approximately 0.26 mm may be at the resolution limit of current DSA techniques [6].

The PTA is the most proximal PCA-derived thalamus-supplying artery. It is also referred to as the paramedian thalamic artery [14], the inferior thalamic artery [15] or the paramedian thalamo-subthalamic artery [16] (further synonyms are listed in Table 1). Concerning the thalamus, the territory of the PTA includes the intralaminar nuclei, the medial nuclei, the dorsomedial nuclei, the ventromedial pulvinar and the posteromedial portion of the lateral nuclei [17]. The PTA and its branches may furthermore supply various brain regions including parts of the hypothalamus, subthalamus, substantia nigra, red nucleus, oculomotor nucleus, reticular formation of the midbrain, posterior part of the internal capsule and pretectum [5].

**Fig. 8:**
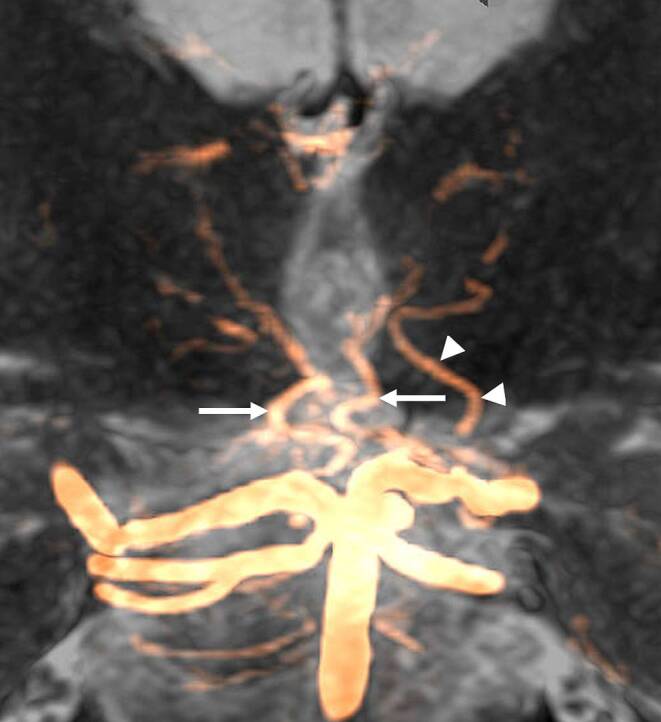
Coronal maximum intensity projection (MIP) of the posterior thalamoperforating arteries (PTA, arrows) and the left anterior thalamoperforating artery (ATA, arrowheads) projected onto a coronal T2 weighted MR image

A commonly used classification of PTA variants has been published by Percheron (Fig. 4; [18]). Type I is characterized by a symmetrical anatomy of the PTAs (Each PTA arises from the corresponding P1 segment and supplying the ipsilateral thalamus, respectively (Fig. 8)). Type II displays an asymmetrical anatomy where paramedian arteries for both thalami originate from the same P1 segment. In subtype IIa, separate trunks are present for the ipsilateral and contralateral PTA, whereas in subtype IIb, a single unilateral trunk provides origin to both the ipsilateral and the contralateral PTA. Type IIb is also known as the “Artery of Percheron”. In type III, both PTAs originate from a shared vascular arcade.

We found a “Artery of Percheron” type IIb PTA in 26% of cases, which is a significantly higher incidence than what was previously documented in cadaver studies (Table 4).

**Table 4:** Reported frequencies of type IIb ATP variants according to Percheron’s classification

Authors	Type IIb (AOP) (in %)	*n*
Uz [19]	7	15
Saeki & Rhoton [20]	8	50
Lang & Brunner [21]	8	50
Pedroza et al. [14]	10.7	56
Park et al. [22]	11.5	26
Kaya et al. [12]	11.7	14
Kocaeli et al. [23]	11.7	34
Griessenauer [24]	12	25
Present study	26	58

*AOP* Artery of Percheron

It has been suggested that AOP may be underdiagnosed due to the significant variation in anatomy and presence of the P1 segment [25, 26]. This also corresponds to our finding that patients with a fetal type ACP origin and ipsilateral small caliber P1 segment never presented with Percheron subtype I.

Instead, the dominant P1 segment appears to be the source of bihemispheric paramedian thalamic supply in these cases. This study’s finding holds clinical significance regarding AOP infarctions. In their recent study, Ciacciarelli et al. [27] reported a frequency of bithalamic paramedian infarctions in 0.53% of stroke patients, and of these occurrences, an AOP was identified in 40%.

However, alternative classifications for PTA origins are introduced by other authors. Uz differentiated four different PTA types in his study [19]. Type I entails multiple branches on both P1 segments. Type II comprises multiple branches arising from only one P1 segment and one or a maximum of two branches arising from the other segment. Type III contains only one large caliber branch on each segment, while Type IV encompasses multiple branches on one P1 segment with no branches on the other.

Neto et al. [28] also categorized PTA variants in in four types. Type I is defined when a large-caliber vessel originates from each P1 segment (according to Percheron’s type I). Type II corresponds to when one trunk arises from each P1 segment and supplies both the ipsi- and contralateral hemisphere. Type III is defined when two trunks originate from each P1 segment and Type IV, when three or more branches originate from each P1 segment.

We adapted the simpler classification proposed by Percheron (Fig. 4). Regardless of the classification used, identifying a variant in which both ATPs originate from a single P1 segment, mainly from a shared trunk, is of high relevance because impairment, such as occlusion of the corresponding P1 segment leads to prognostically unfavorable bilateral median thalamic infarcts [3].

We also identified a higher incidence of subtype III ATPs in our collective as compared to Percheron’s work. Additionally, we found that 15.5% of the ATPs originated from a common, network-like structure—a finding not described by other authors.

It is important to consider the methodological limitations previously mentioned with regards to subtype III and the network-like origin. Narrow caliber vessels may run parallel, overlap, or cross without sharing a common branch or connection. As a result, estimating the distance between arteries using current DSA technology can be challenging. This may potentially have contributed to an overestimation of subtype III in our study.

The PMCA is typically the first group of arteries branching off the P2 segment, also it may originate from the distal P1 segment adjacent to the posterior communicating artery, or arise from one of the PCA branches such as the parieto-occipital, calcarine, or splenial artery [29]. The PMCA consists of one to three branches that supply the tegmentum, including the subthalamic nucleus, parts of the medial geniculate body, the posterior parts of the intralaminar nuclei, and the pulvinar [29, 30]. It penetrates the velum interpositum and forms the plexal segment, which then travels to the roof of the third ventricle. We observed the PMCA in approximately 40% of patients, with multiple branches in 46% of cases. In most cases, the PMCA’s small branches anastomose with the PLCA at the level of the intraventricular foramen of Monro [30].

The TGA comprises of multiple branches that originate from the P2 segment, less commonly from the P3 segment or the PMCA [30]. These branches travel through the ambient cistern and enter the thalamus close to the geniculate bodies, providing blood supply to the lateral and posterior portion of the pulvinar and nuclei of the lateral and caudal thalamus [29, 30]. Since they supply the thalamus, they are also known as the ‘inferolateral arteries’. Ischemic strokes within the TGA territory elicit the syndrome of Déjerine and Roussy featuring chronic neuropathic pain contralateral to the stroke side. Additionally, the syndrome is accompanied by hemianopia, hemiplegia, hemiataxia or hemichoreoathetosis contingent on the size and location of involvement [1, 29].

We identified multiple TGA branches in 67%. In their study, Djulejic et al. [30] observed 2 to 12 TGA separately arising branches, with calibers ranging from 70–580 μm. A common trunk with an average diameter of 320–800 μm was present in 33% of the examined hemispheres. In our study, while we were able to identify multiple TGA branches in the majority of patients, it is important to note that smaller branches may have been missed due to the limited resolution of DSA.

The PLCA branches from the distal P2 segment, laterally to the mesencephalon. It gives rise to thalamic and choroidal branches. The thalamic branches supply the inferolateral region of the pulvinar, the lateral dorsal nucleus, and the lateral posterior nucleus. Some of these branches also supply the lateral geniculate body and medial temporal structures [29]. At choroid plexus level, the PLCA branches anastomose with branches from the PMCA and anterior choroidal artery. The PLCA branches have been reported to vary between one and 5, with an average of 1.6 [30]. As for the thalamus, the inferolateral parts of the pulvinar, the lateral posterior and the lateral dorsal nucleus, and the lateral geniculate body are supplied [7, 29]. The PLCA was readily identifiable in our study due to its easily recognizable anatomical course to the choroid plexus.

As a limitation, our study did not allow for anatomical cadaver correlation given that we evaluated vessels in vivo. It is also worth noting that the investigation did not encompass the posterior communicating artery, which includes the anterior perforating thalamic (tuberothalamic) artery, as in the majority of patients, flow in the posterior communicating artery originated from the anterior circulation. It is moreover important to note that the majority of patients analyzed in our study had vascular pathologies in the posterior circulation. This may have led to an overestimation of the number of PTA variants observed, as neighboring vascular pathologies such as arteriovenous malformations may influence the flow conditions and perfusion of the perforating branches.

As previously noted, the bulk of research regarding the anatomy of the TM vessels, such as the seminal study conducted by Percheron et al. concerning thalamic vessels [31], is based on examinations of cadaver brains. Nonetheless, these methods possess certain limitations. Injecting dye with excessive pressure could compromise the integrity of small vessels, whereas inadequate pressure could impede complete dye penetration into small vascular structures. Regarding narrow-caliber or mesh-like vessel anatomy, this technical challenge could hinder their detection.

Nonetheless, future research should consider in vivo imaging of TM vessels and the assessment of their variability. Improvements in DSA image quality and resolution can increase the reliability of small vessel angiography. It has moreover been shown that non-invasive visualization of small caliber vessels using 7 T magnetic resonance time-of-flight angiography is feasible [32], although studies on larger collectives are currently lacking.

In conclusion, routine 3D-RA can display TM vessels, which could be advantageous for planning neuroradiological and neurosurgical interventions, such as treatment of basilar tip aneurysms or PCA stenosis, in which TM vessels are at risk [33, 34].
